# Intestinal perforation due to intestinal and colonic tuberculosis in a patient with HIV, a nearly lethal complication due to lack of adequate treatment and control in a limited resource country, a case report

**DOI:** 10.1016/j.ijscr.2019.09.038

**Published:** 2019-10-01

**Authors:** William Aguayo, Patricio Gálvez, Pablo Acosta, Christian Rojas, Jose Torres, Johan Aguayo, Jonathan Ayala, Byron Camacho, Gabriel Molina

**Affiliations:** a Hospital un Canto a la Vida and Grupo Digeslap Center, Quito, Ecuador; b Universidad de las Américas, Quito, Ecuador; c Pontificia Universidad del Ecuador, Quito, Ecuador; d Hospital un Canto a la Vida, Quito, Ecuador; e Dr. Roberto Gilbert Elizalde Children’s Hospital, Guayaquil, Ecuador; f Pontificia Universidad del Ecuador and Grupo Digeslap Center, Quito, Ecuador

**Keywords:** Tuberculosis, HIV, HIV/TB coinfection, Intestinal tuberculosis

## Abstract

**Background:**

Coinfection involving Human Immunodeficiency Virus (HIV) and *Mycobacterium tuberculosis* are particularly problematic in resource-limited countries. When both pathogens are present, they accelerate the deterioration of immunological functions in the patient leading to premature death if left untreated. A close follow-up is essential in these high-risk subjects, as inadequate healthcare usually leads to further complications.

**Case presentation:**

We present a case of a 26-year-old woman with a past medical history of HIV infection. Nonetheless, she lacked adequate treatment and did not have sufficient medical supervision to control her disease. She presented to the emergency department with an acute abdomen and multiple bowel perforations that demanded intestinal resection. TB/HIV coinfection was detected and a final diagnosis of bowel perforation due to TB was established.

**Conclusions:**

A high index of suspicion is essential when approaching patients with HIV and acute abdominal pain. A thorough clinical history examination including past medical history, HIV/AIDS (Acquired immunodeficiency syndrome) progression status, and a careful clinical exam are paramount to an early diagnosis and timely medical treatment.

## Highlights

• HIV and TB, are a lethal combination in resource-limited countries without adequate follow up.
• Intestinal perforation due to TB in an HIV patient is a rare complication and depends on the immune status of the host.
• If non-compliance is anticipated when treating TB and HIV, fully supervised therapy should be initiated.

## 1 Introduction

Tuberculosis (TB) is a severe infectious disease responsible for 1.3 to 1.5 million deaths per year since 2014 [1,2]. Although its lethality has seen a decrease worldwide thanks to antibiotic therapies, it continues to carry a significant burden in developing countries [1]. One of the key risk groups for TB are patients infected with Human Immunodeficiency Virus (HIV). Mortality rates, opportunistic infections and rare complications from TB have been shown to increase in individuals carrying HIV [3,4]. Gastrointestinal involvement of TB can result in stricture formation, intestinal obstruction, fistulas, volvulus, and perforations. Intestinal perforation is uncommon but can be a serious and life-threatening complication when it occurs. Early recognition of abdominal symptoms and timely intervention are essential to minimize morbidity and mortality [4].

In resource-limited settings, prevention and consecutive controls are of paramount importance, as the previously described complications could be fatal even with adequate treatment. The attitude of healthcare providers towards HIV and TB patients must change to improve their prognosis. Understanding the social, cultural, political and economic issues that these diseases cause on an individual is essential to improve their care and quality of life overall.

This work has been reported in line with the SCARE criteria [14].

## 2 Case report

Patient is a 26-year-old female with a past medical history of HIV. She was infected at age 15 and showed poor adherence to antiretroviral medication. She reported the combined use of Tenofovir, Emtricitabine, and Efavirenz intermittently, mostly because she did not seek adequate healthcare due to feelings of shame about being associated with the disease. She also missed most of her follow-ups in the last year, and had no CD4 counts or viral load tests performed in over 6 months. She also suffered from intermittent episodes of coughing, bloody stools, and weight loss during this time; however, she didn't seek any medical attention.

She presented to our emergency room with a 10-day history of intermittent lower abdominal pain, nausea, biliary vomits and asthenia. Approximately 24 h prior to admittance, the pain became worse. On clinical examination, a dehydrated, hypotensive and tachycardic patient was encountered. Her abdomen was diffusely tender, and the pain became more intense on touch. Laboratory exams revealed mild leukocytosis and neutrophilia. An arterial gasometry revealed metabolic acidosis with hyperlactatemia.

**Fig. 1:**
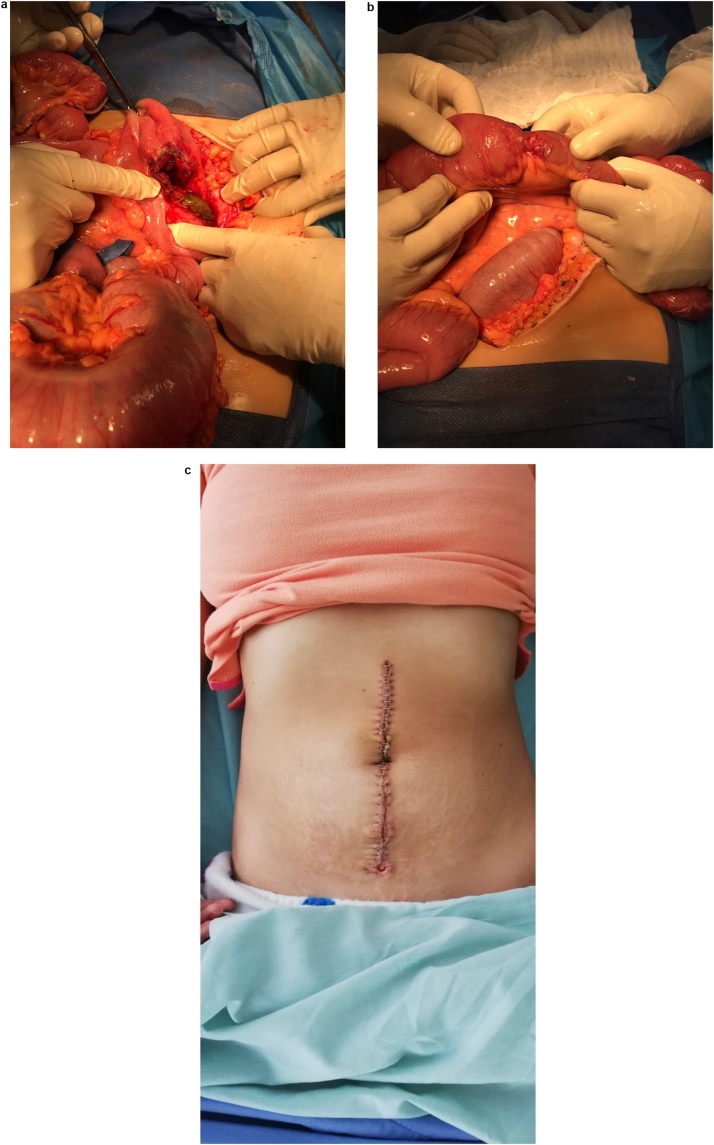
A: Bowel perforation at the cecum. B: Bowel perforation at the terminal ileum. C: Laparotomy wound at the postoperative period.

In view of these findings, surgical consultation was requested and a diagnosis of diffuse peritonitis was reached. Aggressive fluid resuscitation with crystalloids was started and an emergency laparotomy was decided. At surgery, the peritoneal cavity was filled with 500 ml of pus and 2 bowel perforations were identified: a 1 × 1 cm perforation in the terminal ileum, 50 cm proximal to the ileocecal valve, and a 3 × 2 cm perforation in the cecum (Fig. 1A–C).

A right colectomy was decided, however, the surgical team had doubts about restoring bowel continuity. Nonetheless, since the patient became stable after reanimation and surgery, restoration was considered feasible. Given the context of an HIV patient with poor controls who would be unlikely able to handle an ileostomy, a primary anastomosis with close surveillance was decided. Resection began 10 cm proximal to the ileal perforation and ended near the hepatic flexure of the colon. Then, a side-to-side ileotransverse anastomosis was performed with 75 mm staples (ETHICON Linear Cutter NTLC75, Johnson & Johnson). Extensive washing of the peritoneal cavity was completed and the remainder of the procedure was performed without complications. Based on the historical, clinical, and laboratory data obtained, we initially considered intestinal perforation due to cytomegalovirus (CMV) infection as the probable etiology of peritonitis, and the differentials included, mycobacterial infections, fungal infections, neoplastic disease or Kaposi sarcoma. However, definitive pathology tests for the cause of the perforation were delayed due to the unavailability of an in-house pathologist. Furthermore, other essential tests including CD4 counts and HIV viral loads and antibodies (both IgM and IgG) were not available at our institution.

This situation was further complicated by the lack of resources from the patient and our hospital; nonetheless, we managed to send a blood sample to a nearby hospital for testing. Even under these harsh conditions, the patient had a good recovery. She was placed under broad-spectrum antibiotics (Piperacillin/Tazobactam) and was given a 3-day cycle of Ganciclovir. Antiretroviral Therapy (ART) was initiated as well. Meanwhile, sips of liquids were initiated on the second postoperative day without complications. She didn't have any episodes of fever, nausea, vomiting or signs of anastomosis leak.

At the 7th postoperative day, the patient requested to leave against medical advice (AMA), she signed the hospital consents and left our facility.

**Fig. 2:**
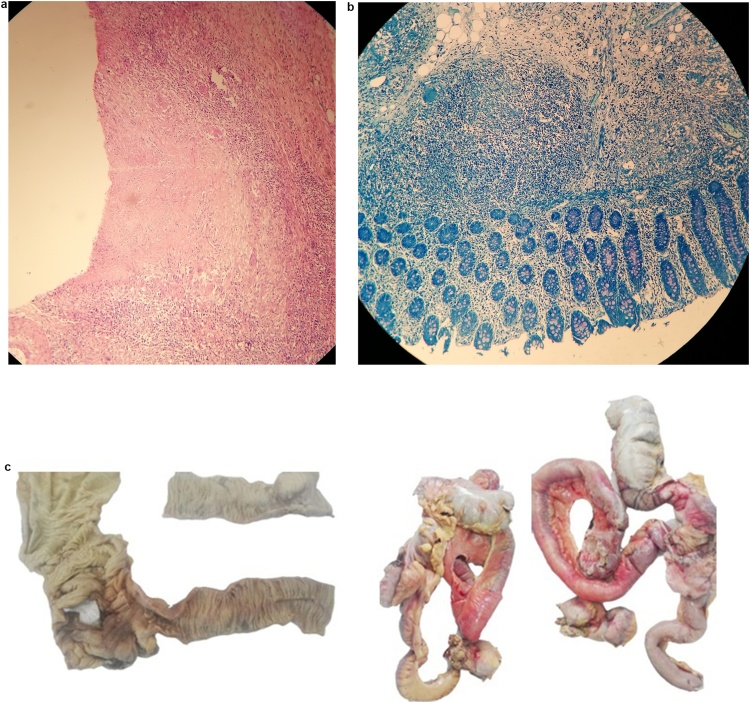
A: H&E stain of TB granuloma, with mucosal ulcer. B: Ziehl-Neelsen stain of a Tb granuloma. C: Resected Bowel.

Two days after the patient went AMA, CD4 and HIV viral load results arrived along with the pathology report, revealing chronic inflammation of the resected bowel, and multiple ulcers affecting the mucosa of the ileum and the cecum. Also, multiple granulomas surrounded by inflammatory tissue were recognized, as well as some granulomas within the lymph nodes. Bowel perforation due to tuberculosis was confirmed as the final diagnosis, CD4 cell counts were estimated at 94 cells/mm^3^, and the viral load was estimated at over 106 genome copies (Fig. 2A–C).

Since tuberculosis in Ecuador is a mandatory declaration disease, we searched for the patient and found her in the coastal region of the country. Fortunately, she was stable and without complications. She was taken by the national healthcare system and admitted to a tertiary hospital for HIV and tuberculosis treatment.

## 3 Discussion

Tuberculosis (TB) is one of the leading causes of death worldwide, and presents the highest death toll of any single infectious agent. In 2017, TB was estimated to have caused 1.3 million deaths, with an additional 1.7 billion people (more than 23% of the world’s population) having latent TB infections, placing them at risk of developing active TB during their lifetime [1]. When TB affects an HIV-positive patient, the co-infection accelerates the deterioration of the patient’s immunological functions and can lead to an early death if left untreated [1,3]. This troublesome scenario is more severe in developing countries, like our own, where the burden of HIV infection is more critical [3].

TB results from infection by a pathogenic bacteria belonging to the *Mycobacterium tuberculosis* complex (which includes several species within the *Mycobacterium* genus). After entering the respiratory tract, these bacilli infect macrophages and in response, CD4 T-lymphocytes produce interferon gamma and interleukin-2, which activate macrophages and cytotoxic cells to try to eliminate the bacilli or delay their intracellular growth. When the immune response is insufficient to limit the growth of the mycobacteria, active TB appears [3,4]. During HIV infection, interferon gamma production and CD4 T-lymphocytes are reduced, which increases the risk of TB infection [[1], [2], [3], [4]]. Likewise, TB also influences HIV progression as cytokines produced by the granulomas worsens HIV viremia, which accelerates the course towards immunosuppression [4,5]. Around 14 million people worldwide are dually affected with HIV and *M. tuberculosis*. These individuals are at greater risk of presenting complications, making TB the leading cause of death among people living with HIV [1,4]. The increased incidence of active TB in HIV infected individuals appear to be caused by the reactivation of a latent TB infection and increased susceptibility for TB infection, although the exact mechanisms of interaction between these two pathogens are still under investigation [3,4]. The clinical presentation varies according to the level of immunity: the common pulmonary illness requires a CD4 cell count higher than 200 cells/mm^3^ [4]. Extrapulmonary infections occur in 9–40% of HIV patients, and are usually secondary to reactivation of a latent infection [5]. Due to its low prevalence and non-specific symptoms, extrapulmonary tuberculosis is difficult to diagnose and control [6,7]. Abdominal TB is the sixth most common form of TB and the commonest type of extrapulmonary tuberculosis in HIV patients [8]. It can affect any organ from the oral cavity to the rectum, and usually develops from the ingestion of contaminated respiratory secretions, hematogenous spread, or contiguous spread from infected organs or lymph nodes. After an initial entry, the mycobacteria infiltrate the intestinal epithelium into the submucosa producing inflammation, ulceration, bleeding, and ultimately perforation [6,7]. The ileocecal region is usually more affected due to more mucosal contact, the effects of digestion and the higher concentrations of lymphoid tissue [5,6]. Symptoms are generally non-specific and can mimic many abdominal pathologies; our patient presented some of the most common symptoms including fever, abdominal pain, night sweats, fatigue, weight loss, constipation, diarrhea, and bleeding [6,8].

Histopathologic examination usually confirms large numerous caseating granulomas in submucosa and serosa with surrounding fibrosis [4,13], a scenario we also encountered.

The treatment for tuberculosis is pharmacological; in HIV patients, TB treatment becomes a public health priority and delays in therapy delivery have been associated with greater mortality rates [9,13]. Our patient didn’t have adequate access to healthcare, making her condition and prognosis more difficult.

Complications are rare and depend on the host immunity and the progression of the disease, and include perforation, bleeding, fistula formation, and obstruction [6,10]. Regretfully, 20–40% of patients will require surgical management [11,12]. In circumstances where surgery is required, the mortality rates range between 14%–50%, and a subsequent course of anti-tuberculous therapy must be employed in order to improve a patient’s survivability [5,13].

The most efficient surgical treatment in perforation cases is the removal of the affected segment with terminal-terminal anastomosis [9,14], a course of action that was followed with our patient.

Since our patient presented with acute abdomen and in critical condition, surgery was considered necessary. Although we suspected tuberculosis, we could not start the treatment without confirmation because it is a disease that is handled exclusively by the national public health system. Fortunately, we had a more adequate follow-up that allowed us to find the patient and complete her treatment.

## 4 Conclusions

The major obstacle in controlling TB and HIV infections in countries like Ecuador is probably non-compliance. If non-compliance is anticipated, fully supervised therapy should be initiated. As HIV patients are at high risk of complications, they must remain under close follow-up controls. As healthcare providers, our perspective must become wider to understand the social, cultural, political and economic issues that contribute and impact the health of an individual. If we can understand the issues that these diseases create in the individual, its family, and its community, we can be better prepared to respond to these health crises and avoid complications through adequate healthcare and family support.

## Sources of funding

The authors have no funding to report.

## Ethical approval

The authors declare that we obtained permission from the ethics committee in our institution.

## Consent

The authors declare that written consent was obtained from the patient before publication of this case.

## Author’s contribution

William G. Aguayo : Conceptualization.

Patricio Fernando Gálvez Salazar : Conceptualization; Data curation; Formal analysis.

Pablo Acosta: Conceptualization; Data curation.

Christian Rojas: Data curation.

Jose Torres: Formal analysis.

Johan Aguayo: Conceptualization; Data curation.

Jonathan Ayala: Conceptualization; Data curation.

Byron Camacho: Conceptualization; Data curation.

Gabriel Alejandro Molina Proaño: Conceptualization; Data curation; Formal analysis.

## Registration of research studies

The authors declare that the patient gave his consent to publish this case, and as this is a case report not human participants were involved in a study.

## Guarantor

Gabriel A. Molina.

## Provenance and peer review

Not commissioned, externally peer-reviewed.

## Declaration of Competing Interest

The authors declares that there is no conflict of interest regarding the publication of this article.
